# TAL Effectors Target the C-Terminal Domain of RNA Polymerase II (CTD) by Inhibiting the Prolyl-Isomerase Activity of a CTD-Associated Cyclophilin

**DOI:** 10.1371/journal.pone.0041553

**Published:** 2012-07-20

**Authors:** Mariane Noronha Domingues, Bruna Medeia de Campos, Maria Luiza Peixoto de Oliveira, Uli Quirino de Mello, Celso Eduardo Benedetti

**Affiliations:** Laboratório Nacional de Biociências, Centro Nacional de Pesquisa em Energia e Materiais, Campinas, Brazil; East Carolina University, United States of America

## Abstract

Transcriptional activator-like (TAL) effectors of plant pathogenic bacteria function as transcription factors in plant cells. However, how TAL effectors control transcription in the host is presently unknown. Previously, we showed that TAL effectors of the citrus canker pathogen *Xanthomonas citri*, named PthAs, targeted the citrus protein complex comprising the thioredoxin CsTdx, ubiquitin-conjugating enzymes CsUev/Ubc13 and cyclophilin CsCyp. Here we show that CsCyp complements the function of Cpr1 and Ess1, two yeast cyclophilins that regulate transcription by the isomerization of proline residues of the regulatory C-terminal domain (CTD) of RNA polymerase II. We also demonstrate that CsCyp, CsTdx, CsUev and four PthA variants interact with the citrus CTD and that CsCyp co-immunoprecipitate with the CTD in citrus cell extracts and with PthA2 transiently expressed in sweet orange epicotyls. The interactions of CsCyp with the CTD and PthA2 were inhibited by cyclosporin A (CsA), a cyclophilin inhibitor. Moreover, we present evidence that PthA2 inhibits the peptidyl-prolyl *cis-trans* isomerase (PPIase) activity of CsCyp in a similar fashion as CsA, and that silencing of CsCyp, as well as treatments with CsA, enhance canker lesions in *X. citri*-infected leaves. Given that CsCyp appears to function as a negative regulator of cell growth and that Ess1 negatively regulates transcription elongation in yeast, we propose that PthAs activate host transcription by inhibiting the PPIase activity of CsCyp on the CTD.

## Introduction

*Xanthomonas citri*, the bacterial pathogen responsible for citrus canker, induces hyperplasic lesions and pustule formation on the host epidermis which result from the increased division and expansion of the plant cells at the site of infection [1]. Global transcriptional analysis of sweet orange leaves challenged with *X. citri* revealed that the bacterium induces major changes in the transcription of genes associated with cell division and growth [2]. Surprisingly, many of the *X. citri*-induced genes, including those encoding cellulases and expansins, involved in cell-wall remodeling, were found to be similarly regulated by auxin and gibberellin, but most importantly, both auxin and gibberellin were shown to be required for initial canker development [3]. Although these data support the idea that *X. citri* promotes cell division and enlargement through changes in the auxin and gibberellin signaling pathways, how exactly the bacterium reprograms transcription in the host is not entirely clear.

It has been shown that the *X. citri* PthA protein, a member of the AvrBs3/PthA family of transcriptional activator-like (TAL) effectors, is not only required for canker elicitation but sufficient to promote cell hypertrophy in citrus leaves [1], [4]–[7]. TAL effectors of the AvrBs3/PthA protein family are translocated into the plant cell by the type-III secretion system and targeted to the nucleus where they function as transcriptional activators [8]. These proteins have the ability to activate transcription in host and non host plants through the recognition of specific *cis*-acting elements located in the promoters of target genes [9]–[12]. The interaction of a TAL effector with its target DNA is mediated by the repeat domain, an internal region of the protein comprising variable, nearly identical tandem repeats of 34 amino acids that define the DNA specificity and determines pathogenicity and avirulence [13], [14].

Previously, we showed that the PthA variants 1–4 from a single *X. citri* strain can form homo and heterodimers. In addition, all PthA variants were shown to interact with the citrus nuclear transporter alpha-importin and to localize to plant cell nucleus [15]. Moreover, structural data obtained for the repeat region of the PthA2 variant (RD2) indicated that this protein domain folds into a tetratricopetide repeat (TPR) superhelix that is structurally related to pentatricopeptide repeat (PPR) motifs known to bind and stabilize mRNAs [16]. The superhelical structure of RD2 was predicted to wrap around the DNA double helix and to undergo compaction upon DNA interaction [16], an idea that was confirmed by recent studies on the three-dimensional structure of the repetitive DNA-binding domain of TAL effectors alone and in complex with DNA [17], [18]. However, despite the advances in the understanding of both the structure and function of TAL effectors, little is still known of how these proteins interact with the host basal transcriptional machinery to activate or modulate transcription.

To address this question, we performed yeast two-hybrid screenings using different PthA variants as baits and identified a number of *Citrus sinensis* (Cs) proteins implicated in protein folding, mRNA stabilization/processing, gene silencing and DNA repair [15], [19]. Among the isolated proteins we started by characterizing a protein complex formed by a cyclophilin (CsCyp), a TPR-containing thioredoxin (CsTdx) and the CsUev/Ubc13 heterodimer involved in K63-linked ubiquitination and DNA repair [15]. Because CsCyp is homologous to ROC1, an *Arabidopsis* prolyl isomerase required for the activation of the bacterial effector protein AvrRpt2 inside the host cell [20], and the mammalian Uev/Ubc13 heterodimer is a component of the U-box ubiquitin ligase CHIP complex [21], we initially hypothesized that the PthA interactors CsCyp, CsTdx and CsUev/Ubc13 might be part of a chaperone complex required for the folding and/or activation of PthAs [15]. However, the fact that recombinant PthA is structured and functional [16] indicates that proline isomerization by CsCyp is not critical for PthA folding or action and that CsCyp may play a different role than that of ROC1.

CsCyp is related to yeast Cpr1, a cyclophilin that regulates gene silencing and controls meiosis trough interactions with the histone deacetylase complexes Sin3-Rpd3 and Set3 [22], [23]. Cpr1 also interacts with and complements the function of Ess1, another prolyl-isomerase regarded as a component of the RNA polymerase II initiation and termination machineries [24], [25]. Ess1 is required for 3′-end formation of pre-mRNAs and transcription termination of small non-coding RNAs, but it also associates with promoter sites and inhibits transcription elongation in yeast [24]–[29].

The mechanism by which Ess1 affects transcriptional machinery involves its peptidyl-prolyl *cis-trans* isomerase (PPIase) activity on the proline residues of the C-terminal domain (CTD) of RNA polymerase (pol) II [25]–[27], [30]. The CTD consists of multiple tandem repeats of the consensus YSPTSPS heptapeptide which play a key role in the transcriptional cycle [31], [32]. The CTD undergoes conformational changes in response to serine phosphorylation and proline isomerization of its YSPTSPS repeats, and the cycling of serine phosphorylation/dephosphorylation and proline isomerization within the repeats control the recruitment and exchange of RNA processing factors that ultimately regulates the progress of transcription [25], [30]–[32]. Given that Cpr1 interacts with Ess1 and with histone deacetylase complexes involved in gene silencing and it becomes essential in yeast cells when the Ess1 function is compromised [22]–[24], we decided to investigate whether CsCyp could play a similar role in the control of transcription through an interaction with the CTD of the citrus RNA pol II.

Here we show that CsCyp not only suppressed the *cpr1* and *ess1* mutations in yeast but interacted with the citrus CTD. Moreover, we found that both PthA2 and the CTD co-immunoprecipitate with CsCyp in citrus cell lysates and that PthA2 inhibited the PPIase activity of CsCyp in a similar fashion as the cyclophilin inhibitor cyclosporin A (CsA). Notably, sweet orange RNAi plants with reduced CsCyp levels produced much larger canker lesions when challenged with *X. citri*, in comparison to normal plants.

Taken together, our data provide the first direct evidence for the concept that TAL effectors influence the progress of transcription through modulation of the activity of CTD accessory proteins.

## Materials and Methods

### Functional complementation of yeast mutants

The cDNA encoding the CsCyp protein [15] was subcloned into the yeast pYEX4T vector (Clontech) for the expression of the glutathione-S transferase (GST)-CsCyp fusion protein upon copper induction. The yeast mutant 33513 (*cpr1Δ::kanMX4/cpr1Δ::kanMX4*) [23] was transformed with the pYEX4T-CsCyp construct or the empty vector (GST control) and the recombinant proteins were analyzed by western blot.

For sporulation assays, cells were first incubated at 30°C for 48 h in pre-sporulation medium (0.8% yeast extract, 0.3% peptone, 6% glucose and 2% agar) and subsequently transferred to sporulation medium (1% potassium acetate, 0.1% yeast extract, 0.05% glucose and 2% agar) for 72 h. Cultures were grown to an optical density of 1.0 at 600 nm and the percentage of asci was estimated by counting the number of asci in the cell population at different time points after transfer of the cells to the sporulation medium. On average, ten microscopic field images chosen randomly from each time point were inspected for the countings.

The yeast wild-type strain W303-1A (*MAT*a *ura3-1 leu2-3*,*112 trp1-1 can1-100 ade2-1 his3-11,15 [psi+]*) and its *ess1^H164R^* mutant derivative [24], transformed with the pYEX4T-CsCyp or the empty vector, were grown in SC-Leu-Ura plates at 30°C for 7 days.

For qualitative analysis of growth in permissive and non-permissive temperatures, liquid cultures were grown to mid-log phase in YPD medium and serial 1∶5 dilutions were spotted onto plates and incubated for 24 h at 21°C or 37°C [24].

### Yeast two-hybrid assays

The DNA fragment corresponding to the C-terminal domain of the *Citrus sinensis* RNA pol II was amplified from the EST EY725107 with oligos CATATGCCTTATGTTGGTGGAATGGCCTTC and GCGGCCGCTTAACGTGAGCTCTTGTCACC and cloned into the *Nde*I/*Not*I sites of pOBD/pOAD vectors [33]. The constructs were verified by DNA sequencing and used as baits/preys in two-hybrid assays, as described previously [15]. Bait (pOBD) and prey (pOAD) constructs, including controls (empty pOBD+pOAD-prey and pOBD-bait+empty pOAD), were used to co-transform *Saccharomyces cerevisiae* strain PJ694a (*MATa trp1-901 leu2-3 112 ura3-52 his3-200 gal4D gal80D LYS2::GAL1-HIS3 GAL2-ADE2 met2::GAL7-lacZ*) [34]. The cells were grown for 5 days at 30°C on SC medium lacking tryptophan (−Trp), leucine (−Leu) and histidine (−His) in the presence or absence of adenine (−/+Ade), and containing up to 5 mM 3-aminotriazole (3AT).

### Protein purification and GST-pulldown assays

The 6×His-tagged proteins including CsCyp, full length PthA2 and its truncations were expressed in *Escherichia coli* BL21(DE3) cells and purified by affinity chromatography, as previously described [15]. After purification, the 6×His tag was cleavage with thrombin at 16°C for 16 hours and used in the pulldown assays.

The CTD was subcloned into the *Sal*I/*Not*I sites of pGEX-4T and expressed in BL21(DE3) cells after isopropyl β-D-1-thiogalactopyranoside (IPTG) induction for 2 h at 30°C. Cell pellets were suspended in phosphate buffered saline (PBS) containing 1 mM dithiothreitol (DTT) and lysozyme. After sonication and centrifugation, soluble fractions of GST fusions were immobilized on glutathione resin and non-bound proteins were removed with three PBS washes. Approximately 50 µM of the 6×His tagged proteins were incubated with the resins containing GST, GST-CTD or GST-CsCyp for 2 h at 4°C in the presence or absence of 150 µM CsA. The beads were washed four times with PBS and the resin-bound proteins were resolved on 13% SDS-PAGE gels. Proteins were transferred onto nylon membranes and probed with the anti-PthA (1∶5000), anti-CsCyp (1∶3000) or anti-GST (1∶3000) sera (Sigma-Aldrich) and developed with the ECL kit (GE Healthcare).

### PPIase assay

The PPIase assay was performed according to Kofron et al. [35]. PPIase activity was measured by the release of the *p*-nitroanilide, which results from the chymotrypsin cleavage of the cyclophilin chromogenic substrate *N*-succinyl-Ala-Ala-Pro-Phe-*p*-nitroanilide (*N*-succinyl-AAPF-*p*-nitroanilide) after its prolyl *cis-trans* isomerization. *N*-succinyl-AAPF-*p*-nitroanilide (Sigma-Aldrich) was dissolved in 470 nM LiCl in trifluoroethanol to maximize the amount of peptide present as the *cis*-isomer. Purified CsCyp without the 6×His tag (∼15 nM) was incubated in the reaction buffer (50 mM Hepes pH 8.0, 100 mM NaCl) and allowed to stabilize at 10°C for 5 min. Purified full-length PthA2, its internal repetitive DNA-binding domain (RD2), its C-terminal domain carrying 5.5 repeat units (5.5 rep+CT), CsA or bovine-serum albumin (BSA), as negative control, were added to the reaction mix to a final concentration of 15 to 30 nM. The reaction started by adding 1 mg of α-chymotrypsin followed by the peptide substrate to a final concentration of 100 µM. The PPIase reaction was monitored at 390 nm for a period of 5 min.

### Protein immunoprecipitation (IP)

Sweet orange epicotyls transiently expressing PthA2 [15] or citrus leaves were macerated in lysis buffer, PBS pH 7.4, 10 mM MgCl_2_, 0.1% Triton X-100, 10 mM ethylenediaminetetraacetic acid (EDTA), containing protease inhibitors (0.1 mM phenylmethylsulfonyl fluoride, 1 mg.mL^−1^ aprotinin, 1 mg.mL^−1^ pepstatin and 1 mg.mL^−1^ leuptin). The cell lysates were immunoprecipitated with the polyclonal anti-CsCyp (1∶50), the monoclonal anti-human RNA pol II, clone 8WG16 (1∶40; Santa Cruz Biotechnology), or with a pre-immune sera as control. Bound proteins immobilized in Protein A beads (PIERCE) were separated by SDS-PAGE and immunodetected with the anti-PthA (1∶3000), anti-CsCyp (1∶3000) or the anti-RNA pol II sera (1∶200).

### Co-localization assays

Nuclear co-localization of PthA and CsCyp was performed in *Nicotiana benthamiana* cells. The constructs expressing the PthA2-GFP and CsCyp-DsRed fusion proteins [15] were each inserted into the *Agrobacterium tumefaciens* strain EHA105. *N. benthamiana* leaves were co-infiltrated with a suspension of both the PthA2-GFP and CsCyp-DsRed-transfected *Agrobacterium* cells, and the infiltrated leaf sectors were visually inspected by fluorescence microscopy, as described previously [15].

### Citrus transformation and RNAi

A DNA region of 502 bp corresponding to the cyclophilin domain of CsCyp was amplified with oligos RNAi-F 5′-TCTAGACTCGAGATGACCGTCGGCGGTCAGCC-3′ and RNAi-R 5′-ATCGATGGTACCCGCAATCAGCGATCACGACGG-3′ and cloned in opposite directions in the pHANNIBAL vector (CSIRO), as described by Wesley et al. [36]. The whole hairpin construct under the control of the 35S promoter was subcloned into the binary pCambia1303 vector (Cambia, Australia). The construct was verified by DNA sequencing and moved into the *A. tumefaciens* EHA105. Etiolated epicotyls of sweet orange ‘Natal’ were transformed with the *Agrobacterium* cells carrying the CsCyp hairpin construct, as described previously [37]. Explants were selected in MS basal medium containing hygromycin (5 mg/L). Selected plants were analyzed by PCR for the presence of the transgene using oligos derived from the 35S promoter and CsCyp hairpin. Transgenic plants were also assayed for the histological beta-glucuronidase (GUS) activity [38]. Leaves of PCR and GUS positive plants were analyzed by western blot using the anti-CsCyp serum (1∶3000).

### Bacterial infiltration and CsA treatment

Six-month-old plants of sweet orange (*C. sinensis*) were obtained from certified nurseries and kept in a green-house with minimum and maximum temperatures of 22 to 35°C and ∼70% relative humidity. *X. citri* strain 306 was grown for 48 h at 30°C on LBON medium containing ampicillin [2]. Cells were suspended in water and counted through serial dilutions and plating. Leaves were infiltrated with approximately 0.1 mL of a *X. citri* suspension (∼2×10^5^.mL^−1^ cells) in the absence and presence of CsA at 0.1 and 0.5 mM final concentrations. Leaves of the CsCyp RNAi plants were challenged with *X. citri* in the absence of CsA.

## Results

### CsCyp complements the yeast *cpr1* and *ess1* mutants

**Figure 1:**
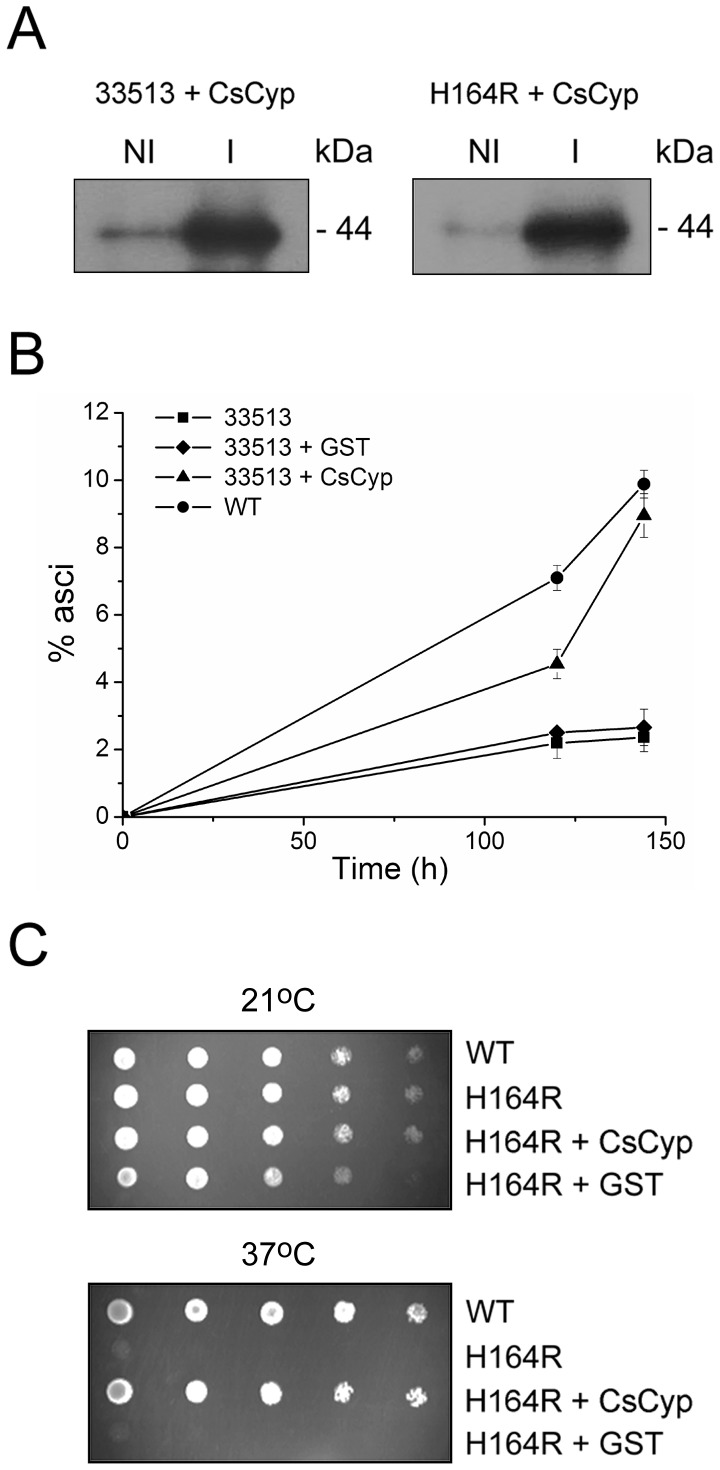
CsCyp complements the *cpr1Δ/cpr1Δ* and *ess1^H164R^* yeast mutants. (A) Expression of CsCyp as a GST fusion in the yeast *cpr1Δ/cpr1Δ* (33513) and *ess1^H164R^* mutants, under the control of a copper-induced promoter. The recombinant proteins were induced with 0.5 mM copper sulfate for 2 h and probed with the anti-GST serum. Induced (I) and non-induced (NI) samples are shown. (B) The *cpr1Δ/cpr1Δ* mutant expressing GST-CsCyp (▴) but not GST alone (♦) increased the rates of sporulation of the 33513 mutant, relative to non-transformed cells (▪). Wild type cells used as reference are indicated (•). Sporulation was measured at different time points after transfer of the cells to sporulation medium and plotted as the percentage of asci formed. Measurements are the means of ten independent countings and the error bars represent standard deviations. (C) Growth of W303-1A wild-type (WT) and *ess1^H164R^* mutant (H164R) in YPD medium supplemented with 0.1 mM copper sulfate at the permissive (21°C) and non-permissive (37°C) temperatures. Cells were grown to the mid-log phase and serial dilutions were spotted onto plates and incubated for 24 h at the indicated temperatures. The growth of the *ess1^H164R^* mutant at 37°C was rescued by GST-CsCyp but not GST alone. Non-transformed WT and *ess1^H164R^* cells served as controls.

Because CsCyp is a nuclear protein [15] and is 64% identical to yeast Cpr1, we tested whether it could complement the *cpr1* mutation, which affects meiosis and sporulation in yeast [23]. CsCyp was expressed in the yeast *cpr1*Δ*/cpr1*Δ mutant [23] as a GST-fusion upon copper induction (Fig. 1A). As shown in Fig. 1B, CsCyp significantly increased the rate of sporulation in the *cpr1* double mutant, compared to GST alone in copper-containing medium, suggesting that CsCyp is the functional homolog of Cpr1. In addition, because Cpr1 physically interacts with Ess1 in yeast and suppresses the *ess1* mutation, we tested whether CsCyp could also rescue the yeast *ess1^H164R^* mutant, which shows cell cycle defects and is compromised in cell viability [22], [24]. Thus, GST-CsCyp was expressed in the yeast thermo-sensitive *ess1^H164R^* mutant [24] upon copper induction (Fig. 1A). Similar to Cpr1, CsCyp suppressed the *ess1^H164R^* mutation and restored the growth of the *ess1* mutant at the non-permissive temperature (Fig. 1C), indicating that its PPIase activity complements that of Ess1. These results therefore suggest that CsCyp could play similar roles in citrus cells as Cpr1 and Ess1 in yeast.

### CsCyp and CsTdx interact with the CTD

**Figure 2:**
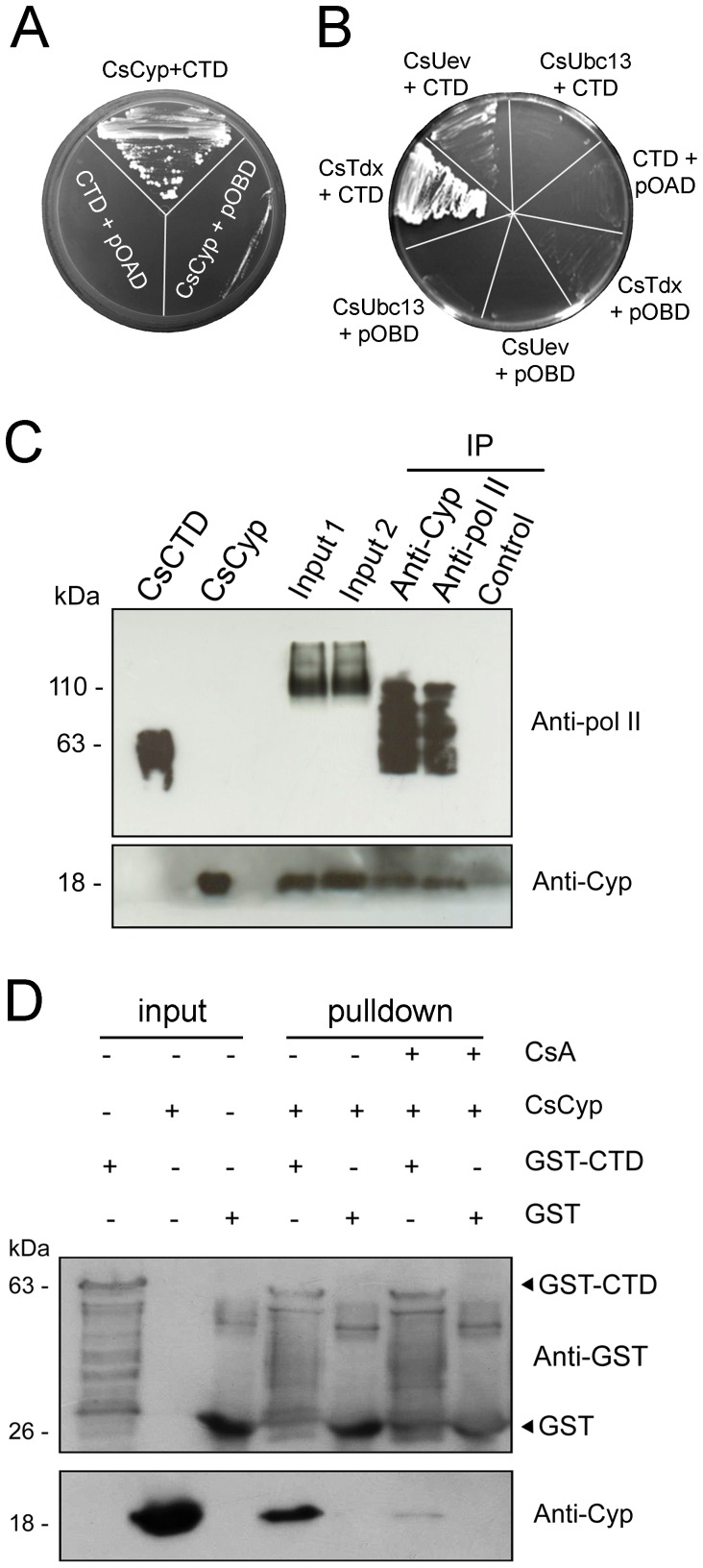
Interactions of the CTD of the citrus RNA polymerase II with CsCyp. (A) Yeast two-hybrid assay showing the interaction between CsCyp (pOAD-CsCyp) and the *C. sinensis* CTD (pOBD-CTD). Control yeast cells co-transformed with the bait (pOBD-CTD) plus empty pOAD or the prey (pOAD-CsCyp) plus empty pOBD are indicated. (B) Yeast two-hybrid assay showing interactions between the CTD with CsTdx (pOAD-CsTdx) and CsUev (pOAD-CsUev), but not CsUbc13 (pOAD-CsUbc13). Yeast cells were co-transformed with the bait plus empty pOAD (CTD+pOAD) or the preys CsTdx, CsUev, CsUbc13 plus empty pOBD as controls. (C) Western blot of immunoprecipitation (IP) reactions showing that the anti-human RNA pol II (anti-pol II) cross-reacted with the recombinant citrus CTD (CsCTD) and with proteins from the citrus cell extracts treated with DNase I (Input 1) or not (Input 2), including a major band of ∼110 kDa. The anti-pol II serum detected various bands in the protein fractions that were immunoprecipitated by the anti-CsCyp and anti-pol II sera, but not by the CsCyp pre-immune serum (control). The anti-CsCyp serum (Anti-Cyp) also detected the recombinant CsCyp and CsCyp that were immunoprecipitated by the anti-CsCyp and anti-pol II sera, but not by the CsCyp pre-immune serum. (D) GST-pulldown assay using the GST-CTD as bait and purified CsCyp without the 6×His tag as prey. Protein samples were electrophoresed and probed with the anti-GST and anti-CsCyp sera. CsCyp bound to the GST-CTD but not to GST alone. The cyclophilin inhibitor CsA prevented CsCyp from interacting with the CTD. Soluble cell extracts of GST-CTD, GST alone and the purified CsCyp used as inputs are indicated and the molecular sizes of the corresponding proteins are shown on the left.

The fact that CsCyp complemented the yeast *cpr1* and *ess1* mutant phenotypes strongly suggested that CsCyp could physically interact with the CTD. To test this assumption, the DNA fragment corresponding to the *C. sinensis* CTD, encoding 36 heptapeptide repeats with the consensus sequence YSPXXPX, was cloned for yeast two-hybrid and GST-pulldown assays. We found that the CTD not only interacted with CsCyp but with CsTdx (Fig. 2A and B), an interacting partner of CsCyp [15]. Interestingly, a weak interaction between the CTD and CsUev, but not CsUbc13, was also noticed (Fig. 2B), indicating that the citrus protein complex comprising CsCyp, CsTdx and the CsUev/Ubc13 heterodimer identified previously as a target of PthAs [15] is associated with the CTD.

To test whether CsCyp associates with the CTD *in vivo*, we immunoprecipitated CsCyp from cell lysates of sweet orange leaves using the anti-CsCyp serum and probed the interacting partners with a monoclonal antibody directed against the largest subunit of the human RNA pol II, which comprises the CTD. As shown in Fig. 2C, the anti-human RNA pol II antibody cross-reacted with the recombinant citrus CTD and detected higher molecular weight bands in citrus extracts suggesting that it recognizes the largest subunit of the citrus RNA pol II. The anti-human RNA pol II antibody detected a minor band of approximately the size of the largest subunit of the citrus RNA pol II (200 kDa) and a major band of ∼110 kDa, which may correspond to a fragment of the RNA pol II largest subunit (Fig. 2C). This ∼110 kDa and other lower molecular weight bands were immunoprecipitated with the anti-CsCyp and anti-human RNA pol II sera, but not with the CsCyp pre-immune serum (Fig. 2C). Although the presence of such multiple bands indicates degradation of the RNA pol II largest subunit during the IP procedure, it is also possible that the anti-CsCyp serum immunoprecipitated other components of the RNA pol II machinery that are recognized by the anti-human RNA pol II antibody. Similarly, CsCyp was detected in the protein fractions that had been immunoprecipitated with the anti-CsCyp and anti-human RNA pol II sera, but not with the CsCyp pre-immune serum, indicating that CsCyp is associated with the CTD *in vivo*.

The interaction of CsCyp with the CTD was further confirmed by GST-pulldown. Most significantly however, this interaction was strongly reduced by the cyclophilin inhibitor CsA (Fig. 2D), suggesting that CsCyp interacts with the CTD through its active site.

### PthA variants interact with the CTD through their LRR region

**Figure 3:**
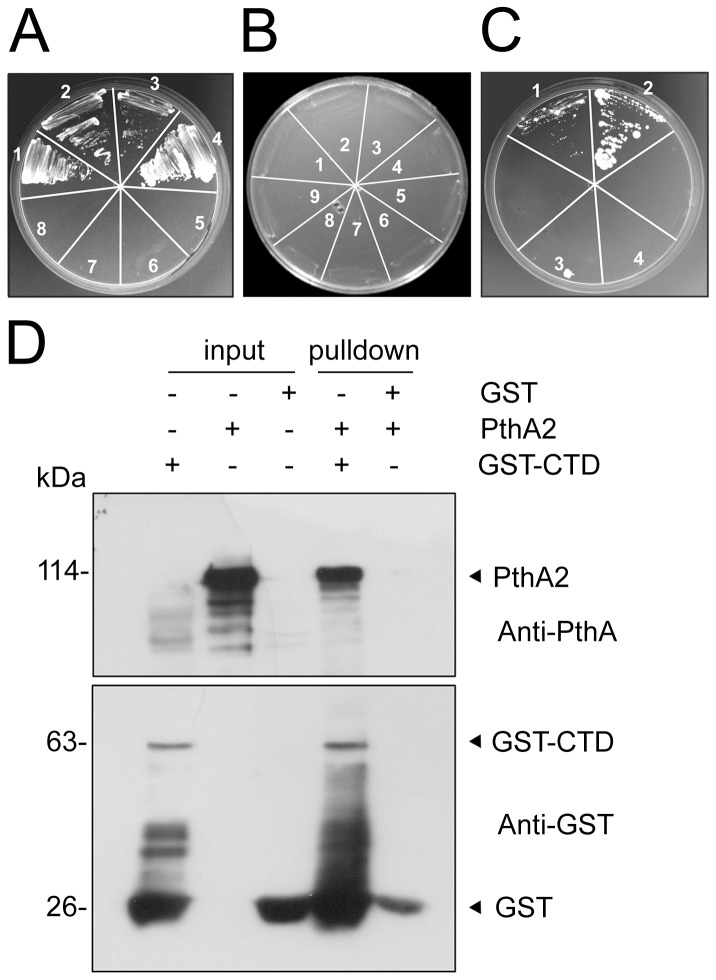
PthA variants interact with the CTD through their LRR region. (A) Yeast two-hybrid assay showing the interaction between the CTD and the four PthA variants (1–4, respectively). Yeast cells co-transformed with the individual pOAD-PthA 1–4 constructs plus empty pOBD (5–8, respectively) were used as controls. (B) No interactions were observed between each of the repeat domains (RDs) of the PthA variants 1–4 (1–4, respectively) with the CTD. Yeast cells co-transformed with the bait pOBD-CTD plus empty pOAD (5) or the preys pOAD-RD 1–4 plus empty pOBD (6–9, respectively), as controls, are indicated. (C) Yeast two-hybrid interactions of the CTD with the truncated version of PthA2 carrying 5.5 internal repeat units plus the C-terminal domain (1) or the LRR region alone (2). Control yeast cells co-transformed with empty pOBD plus the prey constructs pOAD-5.5rep+CT (3) and pOAD-LRR (4) are indicated. (D) GST-pulldown assay using the GST-CTD as bait and purified PthA2 as prey. Protein samples were electrophoresed and probed with the anti-GST and anti-PthA sera. PthA2 bound to the GST-CTD but not to GST alone. Soluble cell extracts of GST-CTD, GST alone and the purified PthA2 used as inputs are indicated and the molecular sizes of the corresponding proteins are shown on the left.

The observation that the CTD interacted with CsCyp and CsTdx led us to investigate whether it would also interact with the *X. citri* PthA variants. Surprisingly, we found that the CTD physically interacts with the four PthA variants in yeast two-hybrid assays (Fig. 3A).

To map the PthA region responsible for such interactions, we performed two-hybrid assays using the repeat regions of the four PthA variants and truncated versions of PthA2 [15] as baits. We found that the variable repeat regions of PthAs, required for DNA recognition, did not interact with the CTD (Fig. 3B). However, the leucine-rich repeat (LRR) domain, which is identical to all PthAs and is located adjacently to the acidic C-terminal transactivation domain, was sufficient for the interaction (Fig. 3C). Curiously, the truncated version of PthA2 carrying 5.5 repeat units plus the C-terminal domain carrying the LRR region interacted more weakly with the CTD than the LRR region alone (Fig. 3C). The interaction between PthA2 with the CTD was also confirmed by GST- pulldown (Fig. 3D).

### PthA2 binds to CsCyp in vivo and co-localizes with CsCyp in the nucleus

**Figure 4:**
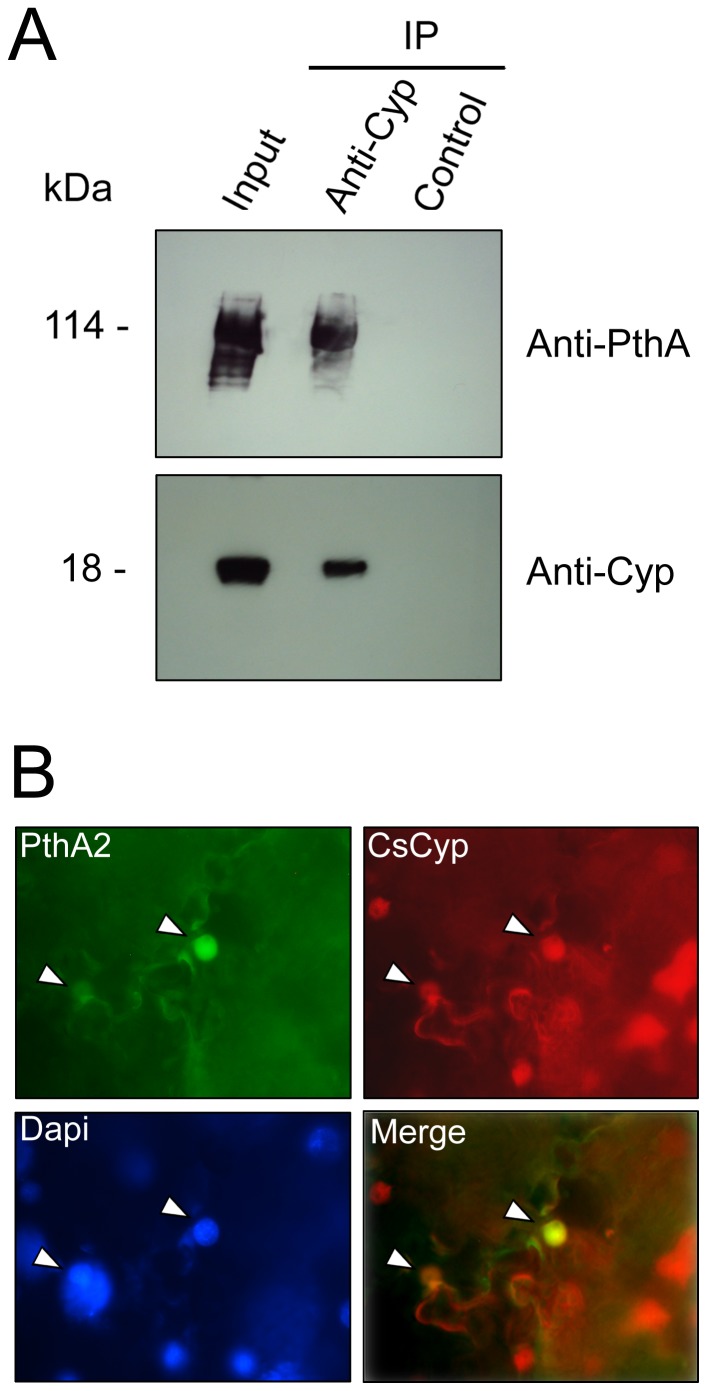
Interaction of PthA2 with CsCyp in plant cells. (A) Immunoprecipitation (IP) assay of PthA2 with the anti-CsCyp serum. Cell extracts of citrus epicotyls transiently expressing PthA2 (input) were incubated with the anti-CsCyp (Anti-Cyp) or the pre-immune serum (control) and protein-A Sepharose. The beads were washed and the bound proteins were resolved on a 13% SDS-polyacrylamide gel and probed with the indicated antibodies. PthA2 was detected in the IP reaction performed with the anti-CsCyp but not with the control pre-immune serum. (B) Co-localization of PthA2 and CsCyp in the nucleus of *N. benthamiana* cells (arrows). Leaf sectors transiently co-expressing PthA2-GFP (PthA2) and CsCyp-DsRed (CsCyp) were treated with Dapi for 10 min before visualization. Pictures were taken in a Nikon fluorescence microscopy at a 1.000× magnification and merged using the software provided by the instrument.

We next investigated whether PthA2 could bind CsCyp in citrus cells. Thus, cell lysates of sweet orange epicotyls expressing PthA2 were incubated with the anti-CsCyp serum and the immunoprecipitate was analyzed by western blot with the anti-PthA serum. Fig. 4A shows that the anti-CsCyp serum immunoprecipitated PthA2 transiently expressed in citrus cells, thereby confirming that PthA2 and CsCyp interact with each other *in vivo*. This result was further supported by the observation that PthA2 co-localizes with CsCyp in the nucleus of *Nicotiana benthamiana* cells (Fig. 4B), corroborating our previous data [15].

### PthA2 inhibits the PPIase activity of CsCyp

**Figure 5:**
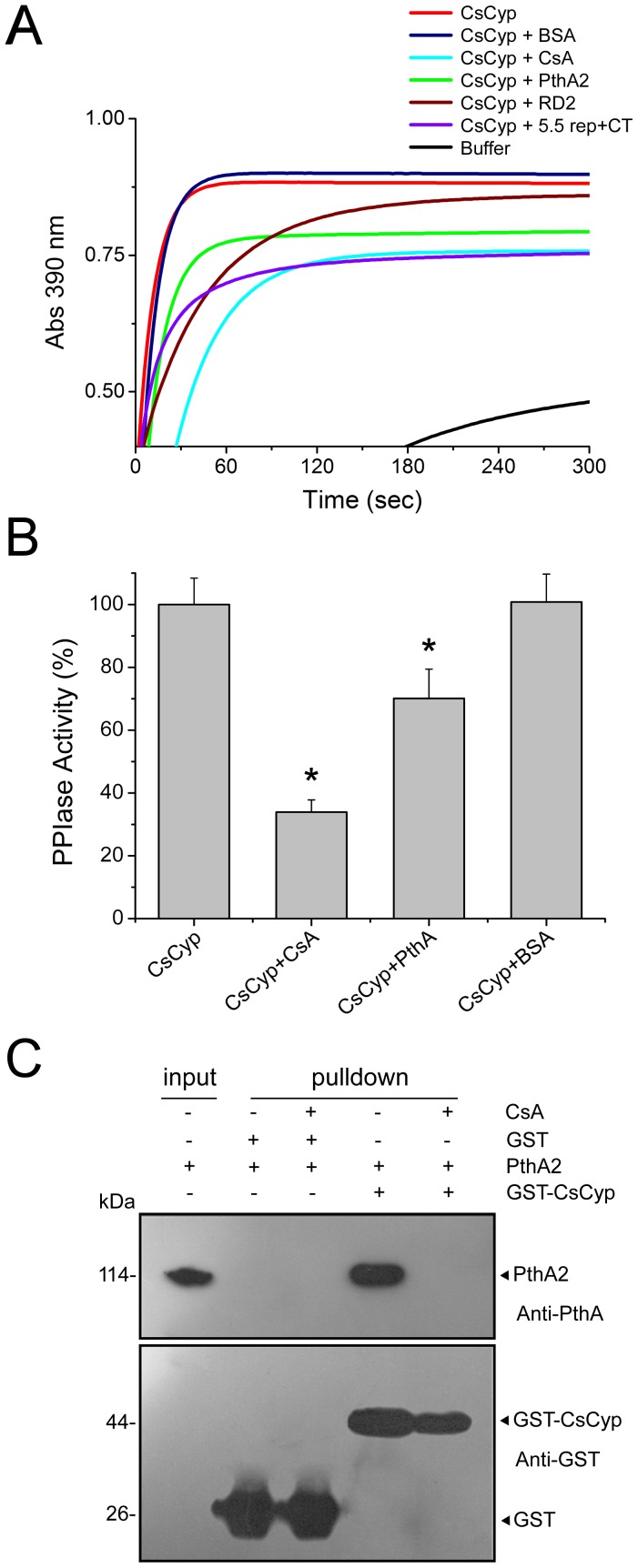
PthA2 inhibits the PPIase activity of CsCyp. (A) The PPIase activity of recombinant CsCyp protein was evaluated by the α-chymotrypsin-coupled assay. Enzyme activity was measured immediately after the addition of α-chymotrypsin and the substrate into the reaction mixture. The PPIase activity of CsCyp reached a plateau within 30 to 60 s after the start of the reaction (red). This activity was drastically reduced in the presence of 30 nM CsA (light blue), as compared to buffer only, as control (black). PthA2 (green), its internal repetitive DNA-binding domain RD2 (brown), or its C-terminal domain+5.5 internal repeats (purple) but not BSA (dark blue), added into the reaction mixture reduced the PPIase activity of CsCyp in a time-course measurement. (B) PPIase activity of CsCyp in the absence and presence of CsA (30 nM), PthA2 or BSA (15 nM). Activities were the mean of three independent measurements recorded 30 s after the start of the reaction, and the asterisks indicate statistically different means relative to that of normal activity. (C) GST-pulldown assay using the GST-CsCyp as bait and purified PthA2 as prey. Protein samples were electrophoresed and probed with the anti-GST and anti-PthA sera. PthA2 bound to the GST-CsCyp only and the cyclophilin inhibitor CsA abrogated the interaction. Purified PthA2 used as input and the molecular sizes of the corresponding proteins are shown on the left.

The interaction of PthA2 with CsCyp suggested that PthA2 might affect the peptidyl-prolyl *cis-trans* isomerase (PPIase) activity of CsCyp. To test this assumption, we first confirmed the PPIase activity of CsCyp by measuring the *cis-trans* conversion and subsequent cleavage of the *N*-succinyl-Ala-Ala-Pro-Phe-*p*-nitroanilide peptide by CsCyp and α-chymotrypsin, respectively [35]. Maximum PPIase activity was observed within 30 to 60 s after the start of the reaction (Fig. 5A). As expected, CsA strongly inhibited the PPIase activity of CsCyp. Surprisingly, addition of equimolar quantities of purified PthA2, but not BSA, into the reaction mixture caused a significant reduction in the PPIase activity of CsCyp within 30 s of reaction. However, while CsA inhibited the CsCyp activity to less than a half, PthA2 reduced it by approximately 25% (Fig. 5B).

To know which domain of PthA2 might be responsible for this inhibitory effect, the PPIase reaction was performed in the presence of the repetitive DNA-binding domain (RD2) or the C-terminal carrying 5.5 internal repeats (5.5 rep+CT) of PthA2. Interestingly, RD2 inhibited the PPIase activity of CsCyp to a much lesser extent than the full length PthA2, whereas the 5.5 rep+CT was apparently more effective than PthA2 to inhibit the CsCyp activity (Fig. 5A). This observation is in line with our previous studies on the interaction of CsCyp with RD2 and 5.5 rep+CT [15].

Given that PthA2 inhibited the CsCyp activity (Fig. 5A and B) and that CsA disrupted the CsCyp-CTD interaction (Fig. 2D), we tested whether CsA could also disrupt the PthA2-CsCyp complex. Indeed, CsA prevented PthA2 from binding to CsCyp in GST-pulldown assays (Fig. 5C), suggesting that PthA2 could also bind to the active site of CsCyp.

### Silencing of CsCyp or CsA treatments enhance canker symptoms

**Figure 6:**
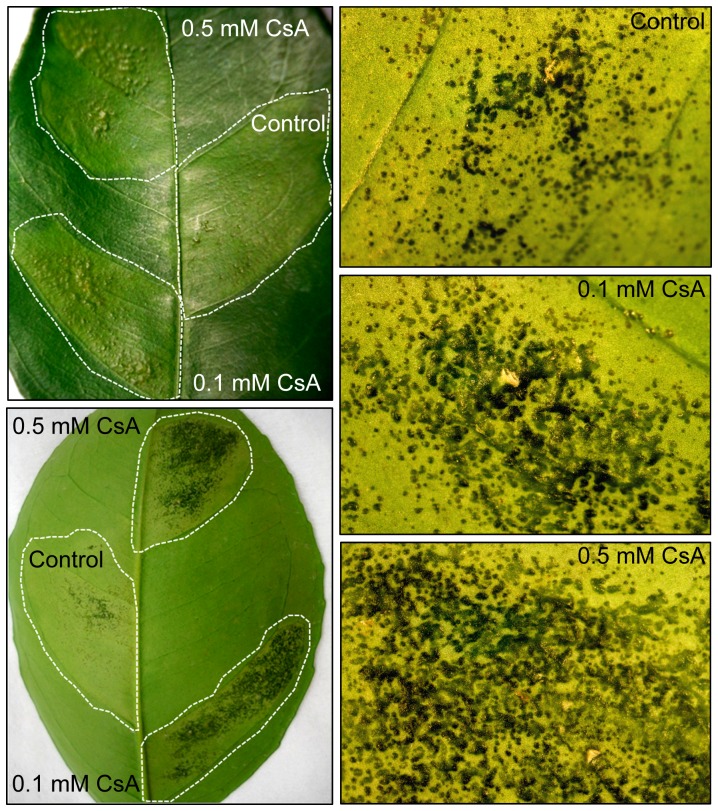
Effect of CsA on citrus canker development. Sweet orange leaves were infiltrated with *X. citri* (approximately 2.0×10^4^ cells) in the absence or presence of CsA at 0.1 or 0.5 mM. Canker lesions started to develop one week after bacterial infiltration and were pronounced 14 days after bacterial inoculation, when the pictures were taken (20× magnification). Left panels show representative leaf sectors that were infiltrated with water suspensions of *X. citri* plus CsA at 0.1 or 0.5 mM final concentrations, or *X. citri* alone, as control (dotted lines). CsA substantially increased pustule formation and symptom development including tissue hypertrophy and water-soaking, relative to control. The effect of CsA on canker development was dose-dependent as 0.5 mM CsA induced more confluent pustules than 0.1 mM CsA or no CsA (right panels).

The data shown above suggested that inhibition of CsCyp by PthA proteins could promote canker development. To test this hypothesis, sweet orange leaves were infiltrated with suspensions of *X. citri* in the presence and absence of CsA. Surprisingly, we found that CsA significantly enhanced canker lesions in a dose-dependent manner (Fig. 6). Leaf sectors infiltrated with *X. citri* plus CsA at 0.1 and 0.5 mM final concentrations showed an increased number of confluent pustules compared to leaf sectors infiltrated with *X. citri* alone (Fig. 6). In addition, pustules from leaf sectors infiltrated with *X. citri* plus CsA developed much faster (10 days after bacterial infiltration) than those infiltrated with *X. citri* only, which typically become pronounced 20 to 30 days after bacteria infiltration. Since CsA alone did not affect the growth of *X. citri* in culture medium or promoted canker in citrus leaves when infiltrated in similar amounts (not shown), our data showing that CsA have a stimulatory effect on canker development supports the idea that PthAs inhibit CsCyp activity *in vivo*.

**Figure 7:**
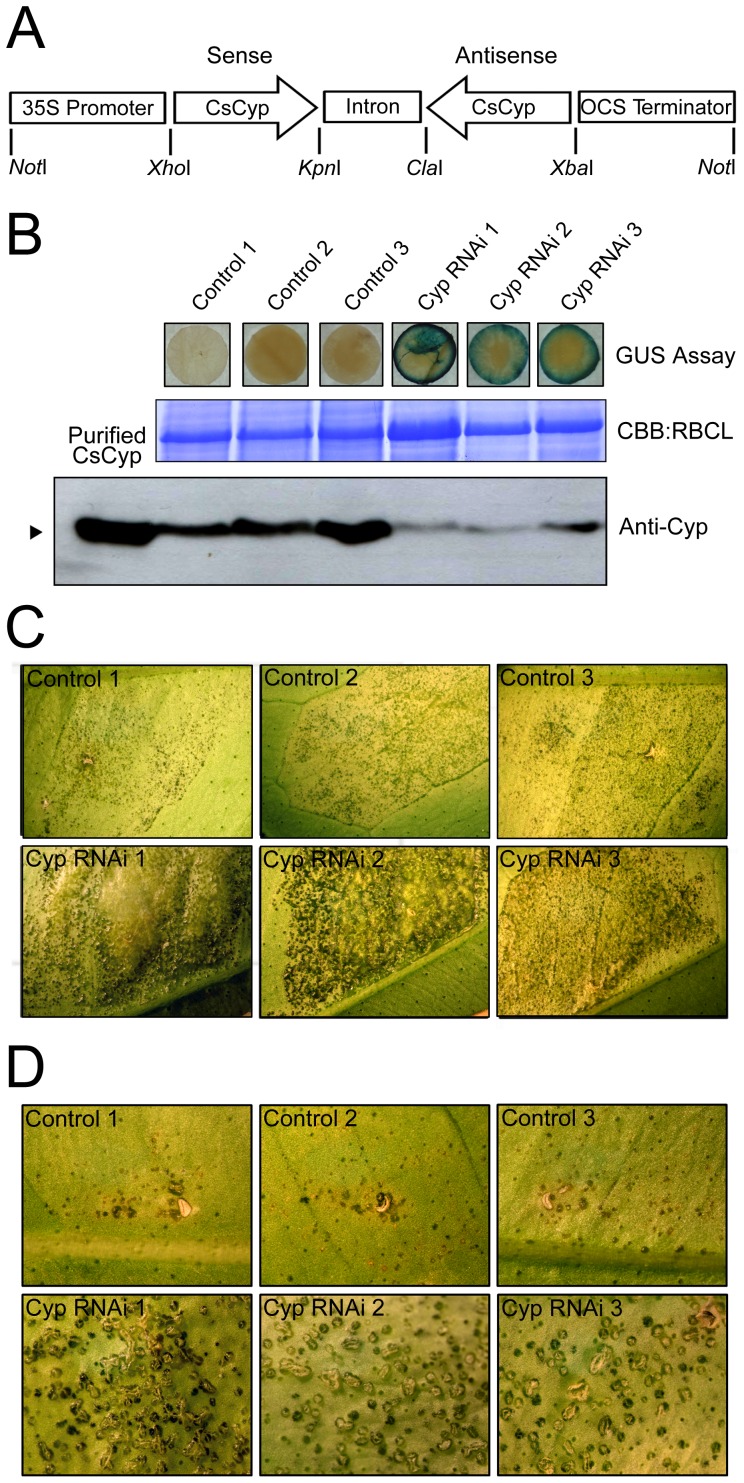
Silencing of CsCyp enhanced canker symptoms. (A) Schematic representation of the RNAi hairpin construct used to transform sweet orange plants. The construct carries the whole cyclophilin domain of CsCyp in an inverted orientation and separated by the intron of the pHANNIBAL vector. (B) Transgenic plants carrying the CsCyp RNAi hairpin construct were assayed for GUS activity and analyzed by western blot using the anti-CsCyp serum (Anti-Cyp). Examples of CsCyp RNAi plants (GUS positive) showing significantly lower levels of CsCyp relative to untransformed controls are shown. Protein loads were controlled by Coomassie Brilliant Blue (CBB) staining of the bands corresponding to the large subunit of Ribulose-1,5-bisphosphate Carboxylase (RBCL). Recombinant CsCyp with no tags was loaded in the first lane of the blot as a positive control. (C) Sweet orange leaves of control and three RNAi plants (Cyp RNAi) challenged with *X. citri* (approximately 2.0×10^4^ cells). Pictures (10× magnifications) were taken 14 days after bacterial infiltration. Canker lesions were substantially enhanced in the RNAi plants compared to controls. (D) Sweet orange leaves of control and RNAi plants infiltrated with *X. citri* at a lower density (approximately 10^3^ cells). Canker pustules in the RNAi plants developed much faster and produced more rupture of the epidermis than in control plants. Pictures (20× magnifications) were taken 20 days after bacterial infiltration.

To further investigate this hypothesis, a CsCyp hairpin construct, comprising the entire cyclophilin domain (Fig. 7A), was expressed in sweet orange plants in order to induce gene silencing [36]. Several transgenic plants carrying the CsCyp hairpin construct were isolated and analyzed by Western blot for the presence of the CsCyp protein. As shown in Fig. 7B, three independent lines of GUS positive RNAi plants showed significantly lower levels of the CsCyp polypeptide than the untransformed control plants, indicating that the hairpin-induced silencing was effective. Although these plants grew normally and showed a phenotype indistinguishable from that of control plants on a visual inspection, they developed more canker symptoms than the untransformed plants when challenged with *X. citri*. Notably, canker lesions in the RNAi plants were substantially enhanced and marked by more confluent, denser and raised pustules (Fig. 7C). Additionally, as observed in the CsA treatment (Fig. 6), canker pustules in the CsCyp RNAi plants developed much faster and produced more rupture of the epidermis than in control plants (Fig. 7D). These results thus show that CsCyp is involved in canker development and functions as a negative regulator or attenuator of cell growth.

## Discussion

**Figure 8:**
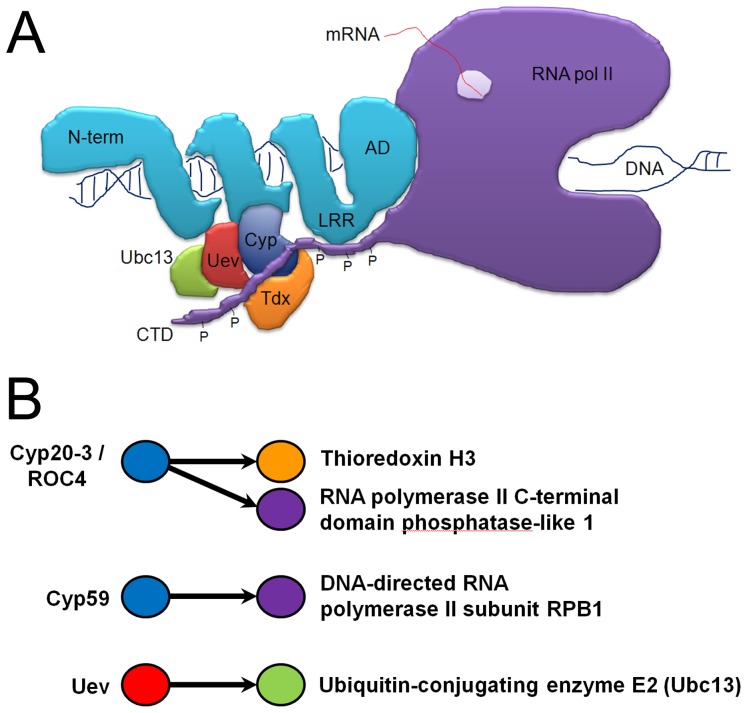
Protein-protein interactions involving cyclophilins and the CTD. (A) Cartoon model of protein-protein interactions illustrating how TAL effectors (PthAs) might associate with components of the host basal transcription machinery. The unfolded CTD of RNA pol II (purple) with phosphorylated heptapeptides (P) may function as a protein scaffold for the assembly of a multiprotein complex, including the citrus CsCyp (blue), CsTdx (orange) and the CsUev/Ubc13 pair (red/green). The N-terminal (N-term), DNA-binding domain, leucine-rich repeat region (LRR) and activation domain (AD) of PthA are shown in light blue. Upon binding to its target DNA, the effector protein associates with the CsCyp/CsTdx/CsUev/Ubc13 complex through its DNA-binding domain [15], and with the CTD through its LRR. The effector protein then inhibits the PPIase activity of CsCyp causing changes in the phosphorylation status of the CTD, allowing the CTD to recruit other co-factors for the progress of transcription. (B) Schematic representation of protein-protein associations found in an Arabidopsis interactome study, depicting the interactions between ROC4 (Cyp20-3) with the CTD and thioredoxin H3, and between Uev and Ubc13 [39]. The Arabidopsis AtCyp59 is also a CTD interactor [40]. Sweet orange and Arabidopsis proteins sharing similar domains are represented by the same colors in A and B.

Although much has been learned recently about the structure, function, target genes and DNA specificity of TAL effectors, the molecular mechanism by which TAL effectors control transcription in the host is still poorly understood. Here, we show that all variants of the effector protein PthA of a *X. citri* pathogen interact with the CTD of the citrus RNA pol II. These interactions appear to be mediated by the invariable LRR region located adjacently to the C-terminal transactivation domain of the PthAs. In addition, we show that the citrus proteins CsCyp, CsTdx and CsUev/Ubc13 heterodimer, identified previously as targets of PthAs [15], are also associated with the CTD. A cartoon depicting such interactions is shown in Fig. 8A. Similarly, protein-protein interactions involving cyclophilins and thioredoxins related to CsCyp/CsTdx were described in an Arabidopsis interactome study [39]. For instance, the prolyl-isomerases AtCyp59 and ROC4 are associated with the CTD whereas ROC4 also interact with thioredoxin H3 [39], [40] (Fig. 8B).

The CTD is known to play critical roles not only in the progress of transcription initiation, elongation and termination, but in the recruitment of numerous cofactors required for chromatin modification, DNA repair and pre-mRNA processing, including capping, splicing cleavage and polyadenylation [29]–[32], [41]–[44]. Surprisingly, in a study to identify specific PthA4 interactors, we found that PthAs also target several proteins implicated in mRNA stabilization/processing such as polyadenylation, translation initiation and elongation factors, as well as proteins involved in RNA cleavage, gene silencing and DNA repair, thought to play roles in mRNA deadenylation and decay [19].

The recruitment of nuclear factors by the CTD is governed by conformational changes in response to serine phosphorylation/dephosphorylation and proline isomerization of its YSPTSPS repeats [25], [30]–[32]. In yeast and mammals, isomerization of the two Ser-Pro peptide bonds of the YSPTSPS repeats, which leads to changes in serine phosphorylation, are performed by the Ess1 and Pin1 prolyl isomerases, respectively [25]–[27], [29], [30], [45]. In yeast, Ess1 interacts with TFIIB and regulates both the early and late stages of the transcription cycle [25]. It also interacts with and is complemented by Cpr1, which in conjunction with Ess1 regulates gene silencing [22].

Previously, we showed that PthAs are not ubiquitinated by the CsUev/Ubc13 heterodimer, but apparently inhibit K63-linked ubiquitination associated with DNA damage induced by methyl-methane sulphonate (MMS), suggesting that the CsCyp/CsTdx/CsUev/Ubc13 complex could be inhibited by PthAs [15]. Considering that i) the *ess1* mutant is sensitive to MMS-induced DNA damage, ii) the Ess1 activity is affected by Rsp5, an ubiquitin ligase that promotes K63 ubiquitination and degradation of the RNA pol II [42], [46], and iii) ubiquitination and degradation of RNA pol II through the CTD occur in response to DNA damage and during transcriptional elongation arrest [42], [43], [47], [48], we proposed that PthAs might activate transcription through modulation of the activity of the CsCyp/Tdx/Uev/Ubc13 complex on the CTD [15]. Here we demonstrate that CsCyp not only complements the function of Ess1 and Cpr1, but interacts with the citrus CTD, which binds PthAs, CsTdx and CsUev. We also show that silencing of CsCyp or treatments with CsA promote canker development and that PthA2 inhibited the PPIase activity of CsCyp in a similar fashion as CsA. These data thus support the notion that by targeting the CsCyp/CsTdx/CsUev/Ubc13 complex, PthAs might affect the stability and/or activity of the CTD. This idea is in line with the observation that i) Pin1 plays a naturally repressive role on transcription, its overexpression causes the release of the RNA pol II from the chromatin, whereas its misregulation is associated with cancer [49]–[51]; ii) Ess1 inhibits transcription elongation and its loss affects transcription termination and cell division [25], [28], [29], [52]; iii) Rrd1, another yeast prolyl isomerase, isomerizes the CTD and promotes the RNA pol II dissociation from the chromatin [53] and iv) ectopic expression of AtCyp59 also resulted in cell growth arrest and decrease in CTD phosphorylation [40].

Furthermore, because AtCyp59 has an RNA recognition motif (RRM) fused to the PPIase domain that binds RNA, it was suggested that AtCyp59 could connect transcription regulation with pre-mRNA processing via its interaction with the CTD [40]. Interestingly, most of the newly-identified PthA4 interactors, which are thought to participate in mRNA stabilization, processing and translation initiation, have single or multiple RRMs [19]. Curiously, the DNA-binding domain of TAL effectors is structurally similar to mTERF [17], [18], a protein known to play critical roles not only in transcription initiation and termination, but in ribosomal biogenesis and translation [54], [55].

Taken together, our results provide strong evidence for the concept that TAL effectors act as transcriptional activators by targeting components of the basal transcriptional machinery that modulate the activity of the CTD and/or stability of the RNA pol II. Although we have not yet been able to obtain citrus plants overexpressing CsCyp, it is possible that by modulating the CsCyp activity canker development can be mitigated.
