# Correction: TAL Effectors Target the C-Terminal Domain of RNA Polymerase II (CTD) by Inhibiting the Prolyl-Isomerase Activity of a CTD-Associated Cyclophilin

**DOI:** 10.1371/annotation/43ed8f9b-0e91-4474-b2bb-fadbd163d650

**Published:** 2013-07-01

**Authors:** Mariane Noronha Domingues, Bruna Medeia de Campos, Maria Luiza Peixoto de Oliveira, Uli Quirino de Mello, Celso Eduardo Benedetti

The name of the second author was incorrectly reflected in the citation. 

The correct citation is: Domingues MN, Campos BM, de Oliveira MLP, de Mello UQ, Benedetti CE (2012) TAL Effectors Target the C-Terminal Domain of RNA Polymerase II (CTD) by Inhibiting the Prolyl-Isomerase Activity of a CTD-Associated Cyclophilin. PLoS ONE 7(7): e41553. doi:10.1371/journal.pone.0041553. 

